# How Noise Can Influence Experience-Based Decision-Making under Different Types of the Provided Information

**DOI:** 10.3390/ijerph191610445

**Published:** 2022-08-22

**Authors:** Youyu Sheng, Di Dong, Gang He, Jingyu Zhang

**Affiliations:** 1 CAS Key Laboratory of Behavioral Science, Institute of Psychology, Beijing 100000, China; 2 Department of Psychology, University of Chinese Academy of Sciences, Beijing 100000, China; 3 Chongqing Changan Automobile Co., Ltd., Chongqing 400000, China

**Keywords:** noise, experienced-based decision-making, experience-congruent suggestion, experience-incongruent suggestion

## Abstract

Pervasive noise undermines many cognitive processes. Across two studies, we examined how noise influences experience-based decision-making and whether the nature of the information provided moderates this influence. Study 1 used the repeated choice paradigm and found that noise can significantly reduce people’s performance in experience-based decision-making by increasing the likelihood of choosing the option with the lower expected value. This negative influence can be attenuated when experience-congruent suggestions are provided, but significantly worsened when experience-incongruent suggestions are provided. Study 2 investigated how noise influences decision-making performance in two experience-incongruent conditions differing in error salience. By replicating noise’s general negative effect, we found that the noise effect could be attenuated when incongruent suggestions were obvious. We suggest that noise can undermine the information updating and integration process, which is necessary for experience-based decision-making. We also discuss the principles for designing better information aids based on these findings.

## 1. Introduction

Noise is pervasive in the modern world. As an important environmental stressor that can continuously undermine human health, it has long been prevalent in factories, mines, airports, military settings, and busy traffic lines. Although great effort has been made to reduce noise in all these places, modern technological development may expose people to some new situations [1]. For example, modern commutation technology can enable mobile working in noisy transportation facilities. The fast deployment of drones may also put people in the noisy environment, even in their daily routine. As a non-negligible environmental stressor, noise directly harms people’s hearing [2]. Repeated exposure to a noisy environment can also harm people’s health through the deterioration of the cardiovascular and neuroendocrine systems (for reviews [3]). Even short-term noise exposure (acute noise) has been found to undermine human cognitive functions including attention, memory, and alertness [4,5,6,7,8,9,10].

Previous studies found that noise has negative effects on human information processing. For example, it can affect attentional abilities [11,12] and can result in poorer performance in working-memory tasks [4,6,10]. Unsurprisingly, noise has also been found to affect learning tasks that require the participation of working memory and attention, such as writing [8], reading comprehension [5], etc.

One possible explanation for noise’s negative effect is distraction. Jones et al. [4] argued that attention could be diverted from the information-processing task (e.g., information retained in working memory) to a noise stimulus unrelated to the task. The effect of distraction also varies with the task characteristics [13]. In addition, noise may increase mental workload and emotional distress, reducing available cognitive resources [14]. Therefore, individuals must either put more effort into completing the task or experience a certain decline in performance in the presence of noise. According to the maximum adaptation theory [15], individuals can adapt to a considerable range of pressures, but task performance is affected once a certain threshold is exceeded. Consequently, noise’s effects increase with intensity and duration.

Furthermore, given noise’s destructive role in cognitive processing, it can be postulated that more complex decision-making is also subject to its influence. However, despite self-reported discomfort in making judgments under noise and the belief that noise may undermine decision-making [16,17,18], hardly any concrete evidence has been found.

For example, Folscher [17] investigated noise’s effects on medical staff’s decision-making. The results showed that, although there was no statistically significant difference in clinical decision-making performance, 65% of participants thought the noise was distracting and 88% reported experiencing some degree of noise-related stress. Lindvall and Västfjäll [16] investigated aircraft noise’s impact on performance and decision-making. Half the participants wore noise-canceling headphones, whereas the other half did not. They then played six internal recordings of Boeing cockpit sounds made during flight. After listening to the recording, participants were asked to rate their emotional responses and how they thought they were performing different tasks. The results showed that participants believed they performed better on the task and made better decisions without noise. Syndicus et al. [18] investigated noise’s effects on risk decision-making among 88 undergraduate students. Four experimental conditions were set up: quiet conditions, office noise (continuous background noise caused by computers, keyboard typing, and occasional ringing phones), a discussion about a museum’s anniversary celebration, and a group discussion about the Ebola virus. The subjects were evenly divided into four groups, and the corresponding sound files were played (participants in quiet conditions were also required to wear headphones). Then, subjects were asked to complete the Lottery Task, the Balloon Analog Risk Task, the Choice Dilemma Questionnaire [19], and the Risk Scenario Questionnaire [20]. After completing the decision-making tasks, the subjects were also asked to evaluate their perception of sound, emotion, and confidence in making decisions. The results showed that the three noise conditions did not affect participants ‘decision-making. However, the participant’s confidence in their decisions was affected when noise was present.

A possible reason behind such findings might be the result of the decision-making paradigm used in their experiments. Descriptive paradigms can best capture the essence of these tasks, which comprise most decision-making research. However, when participants make decisions based on descriptions, they only gather information about the potential outcomes of their choices and the associated probabilities by reading complete abstract descriptions of available options and making once-for-all choices [21,22]. Moreover, because performing this task does not require the continuous activation and updating of attention and working memory, which are the most vulnerable cognitive components in the face of noise, these paradigms can hardly detect noise’s effects.

However, in reality, most decisions are made not only by descriptions but also rely on long-term accumulated experience (e.g., doctors need to make a diagnosis based on their own experience; pilots steered the plane according to their experience). Human factor researchers are particularly interested in these types of tasks, from air traffic controllers that make aircraft maneuvers [23] to firefighters making evacuation plans [24]. Participants must make repeated and continuous choices in a typical experimental paradigm of experience-based decision-making. They may receive no ad hoc information about the possible payoffs of their choices but can only see the results for each turn. Many studies using this paradigm have found that experienced-based decision-making fundamentally differs from description-based decision-making. For example, the framing effect is very salient in a description-based paradigm, which can cause participants to choose the option with fewer expected values (EV) or so-called irrational choices [21]. On the other hand, in the experience-based paradigm, although participants may choose the options by chance at the beginning, they can implicitly learn the actual payoffs of each option and gradually identify the one with the highest EV through repetition [25,26].

In addition, in certain variants of this paradigm, researchers have investigated how experience-based decision-making might be influenced when certain information is presented. For example, Weiss-Cohen et al. [27] found that conflicts with the actual distribution make an important difference. Participants would choose more high-EV options throughout the decision-making process if there were consistent descriptions. However, if there is a conflicting description, in the beginning, they would choose lower EV options in the direction predicted by the misleading information provided. With the accumulation of correct experience information, the ratio of choosing the higher-EV option would increase. This paradigm and related findings have significant implications for designing supportive decision-making systems for human factor researchers. In many information-scarce situations, human operators have to make decisions based on their own experiences. They may also need to rely on the suggestions provided by the decision support systems. When these tools make mistakes, it is difficult for human operators to establish the correct experience base for judgment.

Therefore, this study attempted to follow the paradigm of experience-based decision-making and investigate how the presence of noise could influence it. We suggest that experience-based decisions are undermined by noise, showing three patterns.

First, in contrast to description-based decisions, experience-based decisions rely heavily on the attentive acquisition of new information and continuous updating of working memory [28,29]. Noise can draw participants’ attention toward irrelevant stimuli and undermine memory updating [4,12]. We hypothesized that noise would undermine the overall performance of experience-based decision-making (H1).

Second, as evidence accumulation is a necessary part of experience decision-making, it provides a good opportunity to examine the continuous effect of noise. According to the maximum adaptation theory, noise’s duration increases its negative impact. A longer duration results in accumulated damage to an individual’s cognitive system because it takes time to surpass the adaptation threshold. Therefore, we hypothesized that noise’s negative impact on experience-based decision-making would increase as the duration increases (H2).

Third, when provided the correct information, although participants’ attention is distracted, they would not receive strong enough information to make any changes to their initial choices, so their performance would not be reduced. However, when provided incorrect information about the actual payoffs (experience-incongruence suggestion), the participants need to refute the suggestions to make more favorable decisions. Therefore, they must be very attentive to detect discrepancies between the description and their experience and make a change in their default options. However, when noise is present, it may distract the participants from detecting such discrepancies. As a result, we further hypothesized that noise would be more likely to undermine decisional performance when conflicting information was presented, compared to situations in which experience-congruent suggestions were presented (H3). However, this effect can be attenuated when the discrepancies are more salient (H4).

We conducted two studies to test our hypotheses. Study 1 compared noise’s effects on experience-based decision-making across three conditions (no suggestion, experience-congruent suggestion, and experience-incongruent suggestion). To further test the attentional mechanisms, Study 2 examined whether the noise effect under experience-incongruent information could be reduced when such incongruence was salient to be detected.

## 2. Study 1

### 2.1. Methods

#### 2.1.1. Participants

A total of 154 students were recruited from Tianjin Normal University, including 84 men and 70 women, with an average age of 19.47 (SD = 2.90). All students were right-handed, with no history of neurological disease, color blindness, or color weakness, and had a normal corrected vision. Participants were paid 15 yuan for participating. All participants were recruited publicly through two separate advertisements from a university with a huge quantity of students.

#### 2.1.2. Research Design

This study used a 2 × 3 × 5 mixed design. The first two factors, noise type (noise and no noise) and information type (no suggestion, experience-congruent suggestion, and experience-incongruent suggestion), were between-subjects factors. We followed the block division method of Weiss-Cohen et al. [27] by dividing the 100 trials into 5 blocks (1–20, 21–40, 41–60, 61–80, and 81–100) to better capture the trend of change under different noise by information conditions; therefore, the third factor, time, is a within-subject factor with five levels.

#### 2.1.3. Experience-Based Decision Task

The experienced-based decision task was adapted from Weiss-Cohen et al. [27] and programmed using E-Prime software. Participants were asked to make a series of choices (100 trials) for the two options. They were informed that the two options were fixed to two types of payoffs. They could receive points for each option and were asked to maximize their points from the 100 choices.

The two options had no visual difference but differed in their expected utility and risk. The low-EV option was not risky but had a lower expected utility (100% chance to obtain two points, EV = 2). The high-EV option was riskier, with a higher expected utility (80% chance for five points and 20% chance for 0 points, EV = 4). Although a high-EV option is more favorable, humans‘ risk-averse nature may refrain from choosing it initially [21]. Only after long-term experience would they learn how to choose options with a higher EV, which can be considered a more rational choice [27].

**Figure 1:**
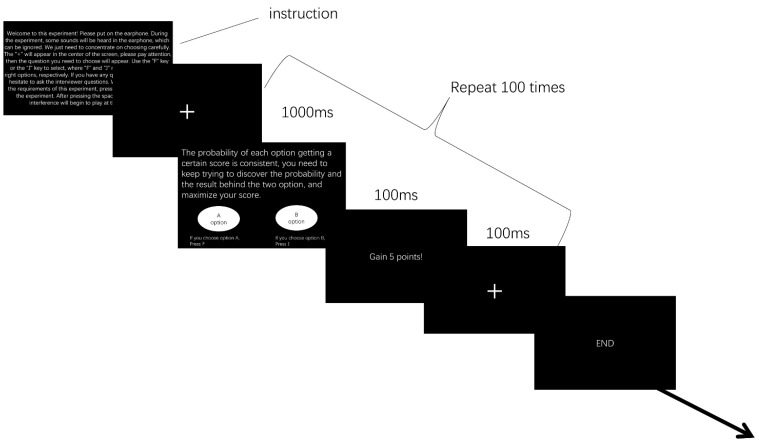
Detailed flowchart of the decision task.

The experiment was conducted on a Lenovo notebook E440 with a 14.4-inch screen and resolution of 1366 × 768 pixels. At the beginning of each trial, a “+” (fixation point) appeared in the center of the screen for 100 ms. Then, the two options appeared with different types of information (see Section 2.1.5). Next, the participants were asked to press “F’ to select the option on the left and “J’ to select the option on the right. The locations of the two options were randomly allocated. After they made a choice, feedback appeared to tell them the actual outcome of their choice (see Figure 1).

#### 2.1.4. Noise Manipulation

All participants experimented with a quiet cubicle to prevent the intervention of environmental sounds and wore SONY MDRE9LP stereo headphones. In the noise condition, participants heard white noise from their earphones and played using Windows Media Player software. The noise had a magnitude of 64.0~66.3 dB(A), and the participants were told to ignore the possible sound played in the headphone when completing the decision task. In the no-noise condition, the participants wore earphones only, and no sound was played.

#### 2.1.5. Manipulation of the Information Type

Along with the two options displayed on the screen, three types of information were provided. In the no-suggestion condition, there was no information about the possible payoffs of the two options. The actual payoffs for the two options were provided in the experience-congruence condition. In the experience-incongruence suggestion condition, the information on the high-EV option was incorrect. The participants were told that this option would have a 20% chance of getting 5 points and an 80% chance of getting 0 points (EV = 1), making this option seem to be less favorable.

#### 2.1.6. Procedure

After arriving at the laboratory, participants were asked to sign an informed consent statement. Subsequently, they were randomly allocated to one of the six between-subject conditions (two noise types × three information types). The participants were asked to wear headphones, start the experimental program, and read the instructions. After confirming that the participants fully understood the experimental requirements, the formal experiment began and lasted for approximately 15 min. When all trials were completed, participants were allowed to remove their headphones; they were thanked, paid, and debriefed.

#### 2.1.7. Data Analysis

The analyses were performed with IBM SPSS Version 22.0 (SPSS, Inc., Chicago, IL, USA).

### 2.2. Results

#### 2.2.1. Preliminary Analysis

Repeated-measures ANOVAs were conducted to examine the influence of noise type, information type, and time on decision outcomes (the percentage of choosing the high-EV option).

**Figure 2:**
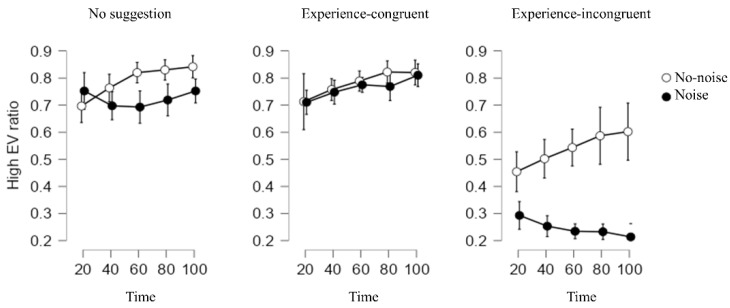
High-EV ratio in each condition of Study 1.

First, we examined how the basic characteristics of experience-based decision-making were replicated. We first performed an information type × time interaction analysis using the three no-noise conditions. The main effect of time was significant, and the performance of the three groups improved over time (1–21: MD = 0.63, SD = 0.27; 21–40: MD = 0.68, SD = 0.25; 41–60: MD = 0.73, SD = 0.23; 61–80: MD = 0.75, SD = 0.24; 81–100: MD = 0.77, SD = 0.26; and *F* (4, 308) = 9.80, *p* < 0.001, *η*^2^*_p_* = 0.036), suggesting the effect of experience accumulation. The main effect of information type was also significant, and the performance of the no-suggestion group and experience-congruent suggestion group was significantly better than the performance of the experience-incongruent suggestion group (no suggestion: MD = 0.79, SD = 0.18; experience-congruent suggestion: MD = 0.78, SD = 0.21; experience-incongruent suggestion: MD = 0.54, SD = 0.13; *F* (2, 77) = 15.29, *p* < 0.001, *η*^2^*_p_* = 0.19). There was no significant interaction between the information type and time (*F* (8, 308) = 0.15, *p* = 0.99, *η*^2^*_p_* = 0.001) (Figure 2).

This analysis replicated the major findings of experience-based decision-making [25] and examined whether the noise-related hypotheses were confirmed. Because the three-way interaction between the noise type, information type, and time was not significant (*F* (8, 592) = 1.49, *p* = 0.17, *η*^2^*_p_* = 0.003), we performed separate analyses in relation to our hypotheses.

#### 2.2.2. Noise’s Negative Effect

We observed a significant main effect of noise (*F* (1, 148) = 13.28, *p* < 0.001, *η*^2^*_p_* = 0.04). In the noise condition, participants were more likely to choose the low-EV option, providing general support for H1.

#### 2.2.3. Noise’s Effect over Time

**Figure 3:**
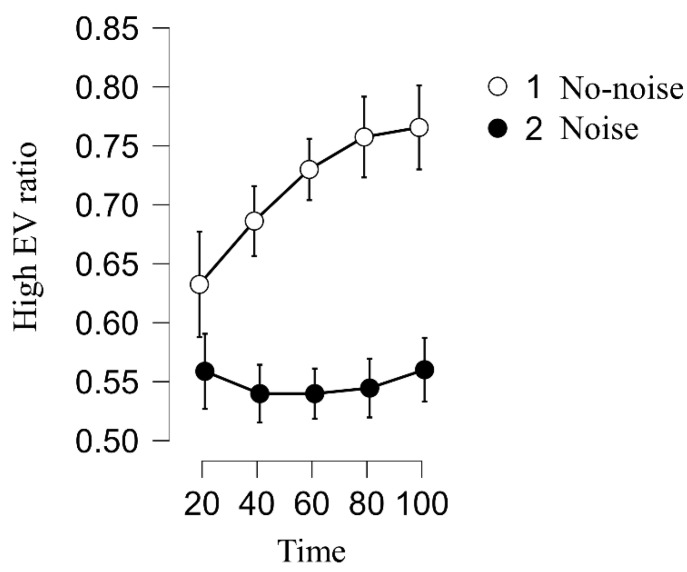
The trend of high-EV ratio in each condition over time of Study 1.

The interaction between the noise type and time was significant (*F* (4, 592) = 6.35, *p* < 0.001, *η*^2^*_p_* = 0.006). Initially, the noise group’s performance was not significantly worse than the control group’s (1–20: *t* = 1.51, *p* = 0.13). However, the difference between the noise group and the non-noise group increased over time (21–40: *t* = 2.96, *p* = 0.003; 41–60: *t* = 3.95, *p* < 0.001; 61–80: *t* = 4.23, *p* < 0.001; and 81–100: *t* = 3.99, *p* < 0.001), Figure 3 depicts the change over time. Thus, the presence of H2 was confirmed. It occurs not only in the experience-incongruent condition (*F* (4, 196) = 4.36, *p* = 0.002, *η*^2*p*^ = 0.02), but in the no-suggestion condition as well (*F* (4, 196) = 4.21, *p* = 0.003, *η*^2*p*^ = 0.02). However, such an effect was not observed in the information-congruent condition (*F* (4, 196) = 0.31, *p* = 0.87, *η*^2*p*^ = 0.001).

#### 2.2.4. Interaction between Noise and Information Type

The interaction between the noise and information type was significant (*F* (2, 148) = 6.03, *p* = 0.003, *η*^2^*_p_* = 0.036). In the no-suggestion condition, there was no significant difference between the noise group’s performance (MD = 0.72, SD = 0.17) and the control group’s (MD = 0.79, SD = 0.22; t = 1.20, *p* = 0.23, *Cohen’s d* = 0.34). In the experience-congruent suggestion condition, there was no significant difference between the control group (MD = 0.78, *SD* = 0.22) and the noise group (MD = 0.76, *SD* = 0.28, *p* = 0.80, *Cohen’s d* = 0.07). However, in the experience-incongruence suggestion condition, the noise group’s performance (MD = 0.25, SD = 0.21) was significantly worse than the control group’s (MD = 0.53, SD = 0.13; t = 5.72, *p* < 0.001; *Cohen’s d =* 1.59). Therefore, H3 was confirmed.

### 2.3. Discussion of Study 1

In this study, we first replicated previous findings on experience-based decision-making. In the no-noise group, regardless of the information condition, participants choose more high-EV options with higher EVs with the accumulation of experience, consistent with the previous study [27].

Then, we made an initial step toward understanding how the presence of noise can influence this process. We found evidence supporting all three of our hypotheses, suggesting that noise may undermine experience-based decision-making, but this influence can be strengthened with time and problematic information provision.

We suggest that the possible mechanisms might be related to the distracted attention caused by noise; however, two alternative explanations must be ruled out. First, this effect can be explained by interference from negative emotions. For example, noise has been shown to affect emotions by making people more irritable [30], which may also undermine their decision-making outcomes, especially when risk is involved [31]. This can also be explained by problems caused by noise that undermines the maintenance or updating of information in the working memory. To further rule out these two possible explanations, Study 2 was conducted.

## 3. Study 2

We conducted Study 2 to replicate the findings of Study 1 further and rule out explanations of emotion and working memory. To understand whether emotion could play a role in this process, we measured participants’ emotions before and after the experiment. Suppose the emotion did not differ after the noise condition or the findings did not change when emotion was controlled as a covariate in the analyses. In that case, we could initially exclude the emotional explanation.

In Study 2, we set up two types of inconsistent information to justify that attention rather than memory maintenance plays a role. Notably, detecting inconsistent information used in Study 1 was difficult. In terms of attention, previous studies found that non-salient stimuli cannot capture attention [32]. However, their performance would improve if we asked the participants to deal with a salient stimulus. Since this would not heavily influence their memory burden, this could be a better test of the attentional influence mechanism we suggested. Thus, we propose the following hypothesis: the noise condition interacts with the salience of conflict; when the conflict is salient, the negative influence of noise is attenuated (H4).

### 3.1. Method

#### 3.1.1. Participants

A total of 153 students were recruited from Tianjin Normal University, including 74 males and 79 females, with an average age of 21.51 (SD = 2.97). All students were right-handed, with no history of neurological disease, color blindness, or color weakness, and had normal corrected vision. Participants were paid 15 yuan for participating. All participants were also recruited publicly through two separate advertisements from a university with a huge quantity of students. The second experiment was conducted 2 months after the finish of the first experiment. There were no overlaps among the two groups of participants.

#### 3.1.2. Research Design

In this study, a noise (two levels) × error salience (two levels) × time (five levels) mixed design was used. The between-subject factor, noise, and the within-subject factor, time, used the same settings as in Study 1. In addition, the new between-subject factor error salience had two levels: salient and non-salient.

#### 3.1.3. The Decision Task

The decision task was similar to that used in Study 1. The difference was in the playoffs and information provided (see Section 3.1.5).

#### 3.1.4. Noise Manipulation

We used the same method for noise manipulation as in Study 1.

#### 3.1.5. Manipulation of Error Salience

Participants in this study were given experience-incongruent information in both conditions. Salience was manipulated to detect experience-incongruent information. In both conditions, the low-EV option was the same as in Study 1 (100% chance of obtaining 2 points). The difference lies in the high-EV option.

In the non-salient condition, the high-EV option had a 75% chance of obtaining five points and a 25% chance of obtaining 0 points. However, the participants were told that this option would have a 75% chance of getting 0 points and a 25% chance of getting 5 points.

In the salient condition, the high-EV option has a 99% chance of obtaining four points and a 1% chance of obtaining zero points. However, participants were told that this option would have a 99% chance of getting 0 points and a 1% chance of getting 4 points.

While the information provided in both conditions was wrong, the participants quickly found that the provided information was incorrect in the salient condition. This is because almost every time they chose this option, the outcome was the opposite of the statement.

#### 3.1.6. Measurement of the Emotion

The Positive and Negative Affect Schedule (PANAS) was used to assess participants’ emotional states before and after the decision-making task. A 5-point Likert scale was used to obtain the scores; the higher the score, the more obvious participants’ mood states. In this experiment, Cronbach’s α coefficients were 0.89 and 0.90 for the positive and negative emotions, respectively.

#### 3.1.7. Procedure

The procedure used in this study was similar to that used in Study 1. The only difference was that the participants were asked to complete the PANAS before and after the decision tasks.

#### 3.1.8. Data Analysis

The analyses performed in this study were the same as that used in Study 1.

### 3.2. Results

#### 3.2.1. Preliminary Analysis

**Figure 4:**
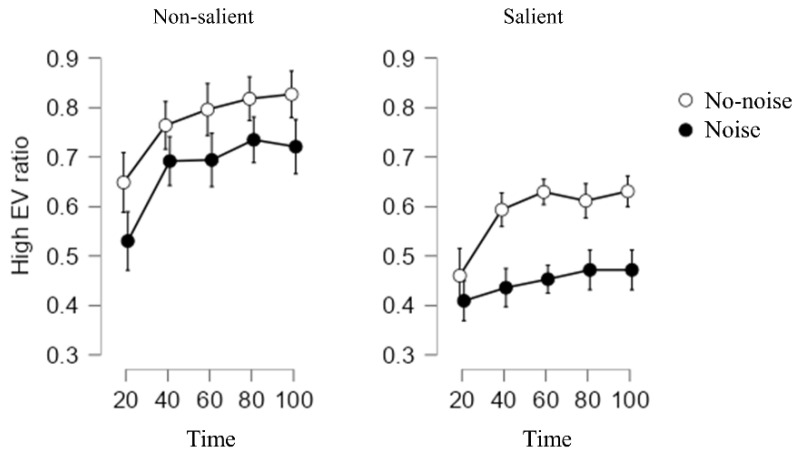
High-EV ratio in each condition of Study 2.

Before the analysis, we standardized the high-EV ratio under each condition owing to the huge difference between the two conditions. Repeated-measures ANOVAs were conducted to examine the influence of noise type, error salience, and time on decision outcomes (the percentage of choosing the high-EV option). As the three-way interaction between noise type, error salience, and time was significant (*F* (4, 596) = 4.07, *p* = 0.002, *η*^2^*_p_* = 0.003; see Figure 4), we conducted separate analyses for our hypotheses.

#### 3.2.2. Noise’s Negative Effect

The main effect of noise was significant (*F* (1,149) = 12.88, *p* < 0.001, *η*^2^*_p_* = 0.06). In both conditions, the noise group’s performance was significantly worse than the control group’s; thus, H1 was confirmed.

#### 3.2.3. Noise’s Effect over Time in Each Condition

In the error-salient condition, the interaction between noise type and time was not significant (*F* (4, 320) = 1.46, *p* = 0.21, *η*^2^*_p_* = 0.0005). The main effect of noise was marginally significant (*F* (1, 80) = 3.85, *p* = 0.053, *η*^2^*_p_* = 0.04), and participants in the no-noise condition were more likely to choose the high-EV option. The main effect of time was also significant (*F* (4, 320) = 186.57, *p* < 0.001, *η*^2^*_p_* = 0.06), and the decision performance was significantly better over time.

In the error-non-salient condition, the interaction between the noise type and time was significant (*F* (4, 276) = 3.54, *p* = 0.007, *η*^2^*_p_* = 0.009). Initially, the noise group’s performance was not significantly worse than the control group’s (1–20: *t* = 0.87, *p* = 0.38). However, the difference between the noise and non-noise groups increased over time (21–40: *t* = 3.20, *p* = 0.002; 41–60: *t* = 3.57, *p* < 0.001; 61–80: *t* = 2.70, *p* = 0.008; 81–100: *t* = 3.22, *p* = 0.002).

Taken together, H2 was only proven in the non-salient condition, but H4 was proved.

#### 3.2.4. Effects of Emotion

Repeated measures ANOVAs were conducted on positive and negative emotion scores measured before and after the decision tasks across the two noise conditions. The interactions between the noise condition and sequence were not significant for either positive or negative emotions (positive, *F* (1, 151) = 0.51, *p* = 0.47, *η*^2^*_p_* = 0.0008; negative, *F* (1, 151) = 2.43, *p* = 0.12, *η*^2^*_p_* = 0.003). Therefore, the noise condition did not induce any emotional changes during the experiments.

We used the changed scores of either positive or negative emotions as covariates to conduct all the above-mentioned analyses. Taking any type of emotional change into consideration did not alter any of the findings that we reported. Interested readers may request analyses by the authors.

### 3.3. Discussion of Study 2

This study first replicated the findings of Study 1, then took a further step to understand how the presence of noise could influence this process. We found evidence supporting our hypotheses in Study 1 and supporting our hypotheses in Study 2, suggesting that when the conflict is salient to detect, noise’s negative influence would be attenuated. Simultaneously, we ruled out explanations for the interference of negative emotions.

## 4. General Discussion

The purpose of the present study was to investigate how noise can influence experience-based decision-making and whether this influence is different when different types of information are provided. In Study 1, participants were offered no information, experienced congruent information, and experienced incongruent information. In Study 2, we further examined possible differences when incongruent information was either salient or not salient. Several findings of this study are worth discussing.

First, we found that noise could undermine the overall performance of experience-based decision-making in both Studies 1 and 2 (H1). This finding contrasts with previous studies using a description-based decision-making paradigm [16,17,18]. As suggested, the reason for this difference might result from the fact that all the information needed to make a description-based decision is fully presented to the participants. However, in experience-based decision-making, which more closely resembles everyday judgment, an option’s real payoffs can only be learned after repeated exposure. During this prolonged process, attentional effort and information updating are heavily involved; because noise has been found to influence these processes, it is reasonable to observe its negative effects.

Second, both studies (H2) found that noise’s negative effect increased as the duration increased. The gradually-worsening effect is consistent with the prediction of maximum adaptation theory [15]. The theory argues that although noise is a negative stressor, it may not have a prompt effect. Individuals can adapt to a considerable range of pressure; cognitive performance becomes undermined only after a certain threshold is exceeded. In the case of noise, a certain amount of time is required for the negative effect to exceed the adaptive threshold.

In addition, while a decreased performance was observed in the experience-incongruent condition and no suggestion condition, there are no differences in the experience-congruent condition. Therefore, according to “the framework of attentional reduction and maximum adaptation theory of noise”, noise could affect the accumulation of the experience information as it may distract attention and undermine the working memory function. However, this is not a problem when the description is congruent with the experience. This is because even when they are distracted from forming a correct experience evaluation, they can still use the pre-informed description (which can truly reflect their actual experience, the congruent condition) to make a proper judgment. However, when there is no pre-informed description (no suggestion) or the pre-informed description was wrong (the incongruent condition), to make a correct judgment, participants must do a heavy job of memorizing and examining their experience or even make a comparison between what they experienced and pre-informed. Therefore, anything that could undermine the accumulation of experience (such as noise) would affect their decisions.

Negative emotions arising during prolonged noise exposure cannot explain this effect. We did not find any changes in emotions due to the presence of noise and taking emotion into account in the analyses did not alter the major findings of this study.

Finally, we found that the noise effect was attenuated when erroneous information was easily detected (H4, Study 2). This finding deepens our understanding of the boundary conditions of noise in experience-based decision-making. Whereas noise may distract people’s attention by undermining their ability to detect discrepancies between provided information and experienced reality, if the discrepancies are sufficiently salient, people are still able to detect them and make a corresponding change in their behaviors. However, even when this happens, people in the noise condition still perform significantly worse than their counterparts in the non-noise condition (see Figure 3). This suggests that the combination of noise and erroneous information has a very problematic effect that is difficult to eliminate.

This study is perhaps the first to demonstrate noise’s negative effect on experience-based decision-making. We believe that this influence was caused by its negative influence on attention. The distraction of noise may undermine the information updating process, which is very important for experience-based decision-making but maybe less so for description-based decision-making. This is also one reason why there is no significant negative effect of noise in previous studies of description decision-making [16,17,18]. We also observed that this effect could worsen over time and with hard-to-detect erroneous information, which can also be explained by the framework of attentional reduction and the maximum adaptation theory of noise. Future studies are needed to replicate these findings and expand them to more comprehensive types of noise (e.g., different intensities, frequencies, and durations) and experience-based decision-making tasks. It would also be interesting to test whether such an effect could be attenuated when participants were more experienced and/or had a higher level of motivation.

This study has several important practical implications. First, it again shows the importance of reducing noise in work settings, especially while learning the patterns of a phenomenon and making repeated decisions. For example, it is worth attempting to reduce environmental noise when maintenance workers fail to sharpen their skills effectively. Second, the noise’s accumulation effect suggests that if noise exposure is unavoidable, the exposure time for certain tasks might need to be reduced. Third, providing task performers with information is essential under noisy conditions. However, sometimes, frontline operators are given incorrect instructions or rely on unsuitable plans, maps, blueprints, or handbooks. In such situations, they must be aware that noise might hinder their ability to identify possible problems. Information tools designed to mark and visualize discrepancies between the provided information and experienced reality might be useful in these situations.

This study has several limitations. First, we only examined the effect of a specific type of noise on one experience-based decision-making task among college students. Younger generations are proficient multitaskers (thus supporting higher numbers of distractors like noise). In our study, the noise’s negative effect is still significant. Therefore, the noise’s negative effect might be more significant in other participant groups who are not proficient multitaskers. Second, although we believe that the noise effect may result from undermined attention, another possible explanation for undermined working memory cannot be ruled out.

## 5. Conclusions

Across two studies, we found that moderate noise can undermine the performance of experience-based decision-making among young adults who are believed to be good at supporting higher numbers of distractors such as noise. This negative effect worsened as the duration increased and the participants provided experience-incongruence suggestions. Moreover, the situation can worsen if erroneous information is challenging to detect. We suggest it resulted from the distraction effect of noise, which might undermine the information updating process.

Future researchers could test whether these findings can be generalized to other types of noise, tasks, and participants. For example, to guarantee repeatability and help identify which group is more vulnerable. Then, future studies may benefit from using an ecological paradigm or physiological apparatus (e.g., EEGs, fMRI, or fNIRS) to understand further the inner mechanisms by which noise affects decision-making. Finally, researchers and practitioners could find ways to improve and train, pay attention to the possible forms of decisional tasks that might be vulnerable to noise, and find effective ways to reduce this impact by developing new training methods.

## Data Availability

The datasets used or analyzed during the current study are available from the corresponding author on reasonable request.

## References

[1] Hanninen O., Knol A.B., Jantunen M., Lim T.A., Conrad A., Rappolder M., Carrer P., Fanetti A.C., Kim R., Buekers J. (2014). Environmental burden of disease in Europe: Assessing nine risk factors in six countries. Environ. Health Perspect..

[2] Basner M., Babisch W., Davis A., Brink M., Clark C., Janssen S., Stansfeld S. (2014). Auditory and non-auditory effects of noise on health. Lancet.

[3] Zaharna M., Guilleminault C. (2010). Sleep, noise and health: Review. Noise Health.

[4] Jones D.M., Macken W.J., Murray A.C. (1993). Disruption of visual short-term memory by changing-state auditory stimuli: The role of segmentation. Mem. Cogn..

[5] Sörqvist P., Halin N., Hygge S. (2010). Individual differences in susceptibility to the effects of speech on reading comprehension. Appl. Cogn. Psychol..

[6] Sörqvist P., Ljungberg J.K., Ljung R. (2010). A sub-process view of working memory capacity: Evidence from effects of speech on prose memory. Memory.

[7] Szalma J., Hancock P. (2011). Noise Effects on Human Performance: A Meta-Analytic Synthesis. Psychol. Bull..

[8] Sorqvist P., Nostl A., Halin N. (2012). Working memory capacity modulates habituation rate: Evidence from a cross-modal auditory distraction paradigm. Psychon. Bull. Rev..

[9] Klatte M., Bergstrom K., Lachmann T. (2013). Does noise affect learning? A short review on noise effects on cognitive performance in children. Front. Psychol..

[10] Sætrevik B., Sörqvist P. (2015). Updating working memory in aircraft noise and speech noise causes different fMRI activations. Scand. J. Psychol..

[11] Broadbent D.E., Harris C.M. (1979). Human performance and noise. Handbook of Noise Control.

[12] Coensel B.D., Botteldooren D., Muer T.D., Berglund B., Nilsson M.E., Lercher P. (2009). A model for the perception of environmental sound based on notice-events. J. Acoust. Soc. Am..

[13] Banbury S.P., Macken W.J., Tremblay S., Jones D.M. (2001). Auditory distraction and short-term memory: Phenomena and practical implications. Hum. Factors.

[14] Becker A.B., Warm J.S., Dember W.N., Hancock P.A. (1995). Effects of jet engine noise and performance feedback on perceived workload in a monitoring task. Int. J. Aviat. Psychol..

[15] Hancock P.A., Warm J.S. (1989). A dynamic model of stress and sustained attention. Hum. Factors.

[16] Lindvall J., Vastfjall D. (2013). The effect of interior aircraft noise on pilot performance. Percept. Mot. Ski..

[17] Folscher L.-L. (2015). Does Loud Noise Affect the Clinical Decision-Making Processes of Healthcare Professionals in a Simulated Emergency Setting?. Ph.D. Thesis.

[18] Syndicus M., Wiese B.S., van Treeck C. (2018). In the Heat and Noise of the Moment: Effects on Risky Decision Making. Environ. Behav..

[19] Kogan N., Wallach M.A. (1964). Risk Taking: A Study in Cognition and Personality.

[20] Rohrmann B. (2011). Risk Attitude Scales: Concepts, Questionnaires, Utilizations Information about Crafted Instruments. http://www.rohrmannresearch.net/pdfs/ras-report.pdf.

[21] Kahneman D., Tversky A. (1979). On the interpretation of intuitive probability: A reply to Jonathan Cohen. Cognition.

[22] Tversky A., Kahneman D. (1992). Advances in Prospect Theory: Cumulative Representation of Uncertainty. J. Risk Uncertain..

[23] Rantanen E., Yang J., Yin S. Comparison of Pilots’ and Controllers’ Conflict Resolution Maneuver Preferences. Proceedings of the Human Factors and Ergonomics Society Annual Meeting.

[24] Klein H.K., Myers M.D. (1999). A set of principles for conducting and evaluating interpretive field studies in information systems. MIS Q..

[25] Barron G., Erev I. (2003). Small feedback-based decisions and their limited correspondence to description-based decisions. J. Behav. Decis. Mak..

[26] Hertwig R., Erev I. (2009). The description–experience gap in risky choice. Trends Cogn. Sci..

[27] Weiss-Cohen L., Konstantinidis E., Speekenbrink M., Harvey N. (2016). Incorporating conflicting descriptions into decisions from experience. Organ. Behav. Hum. Decis. Process..

[28] Ashby N.J., Rakow T. (2014). Forgetting the past: Individual differences in recency in subjective valuations from experience. J. Exp. Psychol. Learn. Mem. Cogn..

[29] Gonzalez C., Dutt V. (2011). Instance-Based Learning: Integrating Sampling and Repeated Decisions From Experience. Psychol. Rev..

[30] Westman J.C., Walters J.R. (1981). Noise and stress: A comprehensive approach. Environ. Health Perspect..

[31] Lerner J.S., Li Y., Valdesolo P., Kassam K.S. (2015). Emotion and Decision Making. Annu. Rev. Psychol..

[32] Gaspelin N., Luck S.J. (2018). The Role of Inhibition in Avoiding Distraction by Salient Stimuli. Trends Cogn. Sci..

